# The Oxford Hip and Knee Scores in patients undergoing hip and knee arthroplasty: cross-cultural validation study based on 110,000 patients

**DOI:** 10.2340/17453674.2026.46545

**Published:** 2026-09-03

**Authors:** Lina H INGELSRUD, Shiraz A SABAH, Eric BOHM, Karl B CHRISTENSEN, Anders TROELSEN, Andrew J PRICE, Anneke SPEKENBRINK-SPOOREN, Anne LÜBBEKE, Christophe BAREA, Jasper MOST, Conrad HARRISON, J Mark WILKINSON, ISAR PROMs working group

**Affiliations:** 1 Department of Orthopaedic Surgery, Copenhagen University Hospital—Hvidovre, Denmark; 2 Department of Clinical Medicine, University of Copenhagen, Denmark; 3 Royal National Orthopaedic Hospital, Stanmore, Oxford, UK; 4 Department of Surgery, University of Manitoba, Canada; 5 Department of Public Health, University of Copenhagen, Denmark; 6 Nuffield Department of Orthopaedics, Rheumatology and Musculoskeletal Science, University of Oxford, UK; 7 Dutch Arthroplasty Register (LROI), The Netherlands; 8 Division of Orthopaedic Surgery and Traumatology, Geneva University Hospitals and University of Geneva, Geneva, Switzerland; 9 Department of Orthopedics, Mobility and Movement, Zuyderland Medical Center, Sittard-Geleen, The Netherlands; 10 Division of Clinical Medicine, University of Sheffield, UK

## Abstract

**Background and purpose:**

Patient-reported outcome measures need to reflect true differences rather than measurement artifacts resulting from translations when scores are compared across countries. We evaluated the measurement invariance of the English, Dutch, Danish, and French Oxford Hip Score (OHS) and Oxford Knee Score (OKS) in patients undergoing hip and knee arthroplasty.

**Methods:**

OHS and OKS responses from patients undergoing primary hip or knee arthroplasty for osteoarthritis between 2019 and 2022 were included from national and single-center registries in the United Kingdom, the Netherlands, Denmark, and Switzerland. We evaluated unidimensionality, monotonicity (Hi > 0.3) and local independence (Yen Q3 > 0.20 above the average indicating independence) in each language version separately. Model fit was considered satisfactory when Comparative Fit Index (CFI) and Tucker–Lewis Index (TLI) were ≥ 0.95, root mean square error of approximation (RMSEA) ≤ 0.06, and standardized root mean square residual (SRMR) ≤ 0.08. Measurement invariance across languages was analyzed with multiple group—confirmatory factor analysis on full, random, and matched sample datasets.

**Results:**

Across the hip and knee cohorts, unidimensionality was acceptable in the English (n = 21,108 and 28,230), Dutch (n = 36,792 and 29,651), Danish (n = 815 and 1,015), and French (n = 590 and 459) versions. RMSEA ranged from 0.072 to 0.092 for OHS, and from 0.057 to 0.061 for OKS and other fit indices were acceptable. The multiple group-confirmatory factor analysis showed acceptable measurement invariance across languages for both scores, with changes in RMSEA < 0.15 and CFI < 0.1 across gradually more constrained models.

**Conclusion:**

Measurement invariance of the OHS and OKS was supported across the English, Dutch, Danish, and French versions, with multiple group-confirmatory factor analyses model fit within predefined acceptable limits, supporting cross-comparison of the different language versions of these instruments.

The Oxford Hip Score (OHS) and Oxford Knee Score (OKS) are 2 well-established patient-reported outcome measures (PROMs) used to evaluate hip and knee health (combined pain and function) in patients undergoing hip or knee arthroplasty [1-3]. The OHS and OKS have been translated into several languages and found to have good validity, reliability, and responsiveness [4-11]. Furthermore, OHS and OKS are the most used joint-specific PROMs in hip and knee arthroplasty registries worldwide [12]. The ability to compare delivery of and outcomes following hip and knee replacement across different populations is important for understanding the effectiveness of these procedures. However, performing head-to-head comparisons of PROM results across countries is not without pitfalls [13]. Differences in PROMs between populations may reflect true differences (e.g., more severe disease at presentation resulting in worse hip or knee health), cultural differences (e.g., kneeling may be more important to some groups than others), or measurement artifacts resulting from translation, leading to different response patterns [14].

Despite the established use of the OHS and OKS instruments, it is uncertain how well these scores compare across language versions. New language versions have been developed using recommended forward- and back-translation procedures; however, stringent evaluations of scale equivalence across language versions have not been performed. This creates uncertainty concerning the comparability of treatment outcomes when measured using the OKS and OHS across registries and countries. When comparing PROMs across languages, different types of invariances can be tested, including semantic, item-level, and scale-level invariance [15]. Scale-level invariant PROMs allow us to meaningfully interpret observed total score differences as reflecting true differences rather than measurement bias.

The aim of our study was to evaluate the measurement invariance of the OHS and OKS scales across different language versions in patients undergoing hip or knee arthroplasty.

## Methods

### Study design

A psychometric evaluation of the cross-cultural validity of the OHS and OKS was undertaken, following the COnsensus-based Standards for the selection of health Measurement Instruments (COSMIN) guidelines [14].

### Participants

Data were obtained through 4 selected arthroplasty registries in which the OHS or OKS are used, representing 4 different language versions: (i) English—the United Kingdom National Joint Registry (NJR) that sources the PROMs data from the National Health Service England PROMs program (NHS PROMs) ; (ii) Dutch—the Dutch Arthroplasty Register (Landelijke Registratie Orthopedische Interventies [LROI]); (iii) Danish—the Copenhagen University Hospital Hvidovre’s local arthroplasty database; and (iv) French (Swiss)—the Geneva Arthroplasty Registry.

The NJR covers arthroplasty procedures across England, Wales, Northern Ireland, the Isle of Man, and Guernsey. The NHS PROMs program was introduced in 2009. The Dutch Arthroplasty Register is also a nationwide registry that initiated the registration of hip and knee procedures in 2007 and PROM collection in 2014. The Geneva Arthroplasty Registry is regional and located at the Geneva University Hospitals, Geneva, Switzerland. The registry was initiated in 1996 (hips) and 1998 (knees), and the PROMs collection was initiated in 2002. The local arthroplasty database at the Copenhagen University Hospital – Hvidovre, Denmark was initiated in 2014. Inclusion criteria were patients undergoing primary elective knee or hip replacement for osteoarthritis (i.e., excluding procedures for trauma, cancer, or rheumatic disorders) between 2019 and 2022, who had answered both preoperative and at least one postoperative questionnaire at either 6- or 12-month follow-up.

### OHS and OKS

The OHS and OKS both contain 12 questions regarding pain and functional limitations due to the hip or knee joint that are answered on 5-point Likert scales [1,2]. Both scales were developed based on classical test theory, in which each response option is equally weighted, and a total score is simply summed based on the response option scores from 0 to 4, giving a total score that ranges from 0 (worst hip/knee health) to 48 (best hip/knee health) [16].

### Outcomes

The outcome was cross-cultural validity of the 4 language versions of the OHS and OKS, assessed through measurement invariance analyses. Evaluation of measurement invariance across languages was performed in a stepwise manner. The first step involved evaluating the validity of the scales in each language cohort separately and in the second step we combined these 4 language datasets to evaluate the validity across languages. Structural validity is one element of validity, ensuring that the questions in a questionnaire are grouped together appropriately for measuring the intended construct (i.e., hip/knee health), justifying their combination into a total score [14]. In the case of the OHS and OKS, the 12 item scores are summed into a single index score for each instrument. For this approach to be meaningful, the OHS/OKS questions should reflect a single underlying construct (unidimensionality) [14]. In addition, key measurement assumptions such as monotonicity and local independence should be reasonably satisfied. Monotonicity means that the questions and response options function consistently, such that scores on each item increase with increasing levels of hip or knee health [17]. Finally, item independence should be evaluated to ensure that responses to individual OHS and OKS items are not overly dependent on each other; in other words, they should not be related beyond their shared association with the underlying trait (hip/knee health) [18]. In the first step, we performed these initial structural validity evaluations on preoperative data for each of the hip, knee, and language cohorts separately. Subsequently, cross-cultural measurement invariance across language versions was investigated using pooled datasets.

### Psychometric properties and statistics

Unidimensionality was assessed using confirmatory factor analysis (CFA), testing whether all questions were related to and measured a single underlying construct of hip or knee health. In this analysis, we specified that all 12 OHS or OKS items loaded onto a single factor meaning that each item contributed to the measurement of the same underlying construct [14]. Because the data is ordinal, we used the diagonally weighted least-squares (DWLS) estimator with robust standard errors and mean- and variance-adjusted test statistics (WLSMV). Adequate CFA model fit was defined as a Root Mean Square Error of Approximation (RMSEA) below 0.06, Standardized Root Mean Square Residual (SRMR) below 0.08, Comparative Fit Index (CFI) above 0.95, and Tucker–Lewis Index (TLI) above 0.95 [19]. Items with a Loevinger’s Hi coefficient above 0.3 were considered to reflect monotonicity [17]. Item independence was tested by inspecting the residual correlation using the Yen Q3 critical value of 0.2 above the average to indicate local dependence [18].

The second step of the measurement invariance evaluations involved multiple-group CFA across the 4 language cohorts. Measurement invariance implies that the construct (hip or knee joint health) is measured in the same way in the different language versions [20]. Multiple-group CFA was performed in steps, each adding stricter assumptions across language versions. First, we tested whether the overall structure of the questionnaire was similar across languages (a baseline, configural model). Second, we examined whether people across languages used the response options in a comparable way (by constraining item thresholds). Third, we tested whether each item contributed similarly to the overall score across languages by additionally forcing factor loadings to be the same across languages. Model fit of RMSEA changing less than or equal to 0.015, together with CFI changing < 0.01 between models, were taken as an indication of invariance across the languages, which would justify comparing scores across language versions [15].

Stepwise multiple-group CFA was performed on (i) full datasets, (ii) matched datasets, and (iii) random samples from each dataset, with the sample size determined by the smallest cohort (French [Swiss]), which was included in full. Random sampling and matching resulted in datasets with unselected response options, which were then collapsed with the neighboring response option before running the multiple-group CFA. To generate matched datasets, we performed 1:1 nearest-neighbor propensity score matching without replacement. English, Dutch, and Danish data was matched to the French (Swiss) dataset. The propensity score was estimated using a logistic regression model including age (< 60 vs ≥ 60 years), body mass index (BMI < 30 vs ≥ 30), ASA physical status classification (I–II vs III–IV), and sex (male vs female). Covariate balance before and after matching was assessed using standardized mean differences, with values < 0.10 indicating good balance.

CFA and multiple-group CFA were performed using the “lavaan” package, and matching was undertaken using the “Matchit” package in the statistical software R (R Foundation for Statistical Computing, Vienna, Austria).

### Ethics, registration, data sharing plan, funding, and potential conflicts of interest

The data used for this study included only that collected as part of systematic data collection in national or local arthroplasty registries. This study was approved by the Capitol Region of Denmark Research and Innovation (p-2023-14410). Approvals were obtained from each contributing registry. We are not able to share the data used in this study, as data is owned by the contributing registries. This study was supported by the International Society of Arthroplasty Registries (ISAR) and the Department of Orthopaedic Surgery at the Copenhagen University Hospital – Hvidovre. Several authors are engaged in the ISAR. The authors declare no other potential conflicts of interest related to this study. Complete disclosure of interest forms according to ICMJE are available on the article page, doi: 10.2340/17453674.2026.46545

## Results

We sourced data from 59,305 patients for the OHS analyses and 59,355 patients for the OKS analyses, with the English and Dutch cohorts representing national level datasets being the largest (Table 1). The French (Swiss) patients were the oldest and had the highest proportion of patients with ASA scores of 3 or 4 across all hip and knee cohorts. The Danish cohort had the highest proportion of unicompartmental knee arthroplasties. OHS and OKS preoperative and postoperative total mean score distributions showed the lowest preoperative scores for the English cohorts and the highest preoperative and postoperative scores for the Dutch cohorts (Table 1 and Supplementary Figures S1 and S2).

**Table 1:** Descriptive characteristics of patients included in the 4 language cohorts for the OHS and OKS. Values are count (%) unless otherwise specified

Characteristic	English ^a^	Dutch ^b^	Danish ^c^	French (Swiss) ^d^
**OHS**, n	21,108	36,792	815	590
Year of surgery	2019–2021	2019–2022	2019–2022	2019–2022
Age mean (SD)	69 (9.8)	69 (9.1)	68 (11.2)	70 (11.9)
Female	12,402 (59)	23,245 (63)	462 (57)	322 (55)
BMI mean (SD)	28.7 (5.0)	27.2 (4.4)	27.7 (5.3)	26.9 (5.4)
ASA				
1	2,379 (11)	6,526 (18)	116 (14)	38 (6.4)
2	15,226 (72)	23,218 (63)	523 (64)	386 (65)
3–4	3,503 (17)	7,037 (19)	176 (21)	166 (28)
OHS, mean (SD)				
preoperatively	17.7 (8.0)	23.2 (8.5)	20.8 (8.0)	19.3 (8.4)
valid score, n	20,933	36,792	815	588
6 months	40.7 (8.1)	–	–	–
valid score, n	20,908			
12 months	–	42.6 (6.7)	40.5 (8.5)	41.1 (8.5)
valid score, n		36,792	815	590
**OKS**, n	28,230	29,651	1,015	459
Year of surgery	2019–2021	2019–2022	2019–2022	2019–2022
TKA	24,958 (88)	24,216 (82)	554 (55)	444 (97)
UKA	3,272 (12)	5,435 (18)	461 (45)	15 (3.3)
Age mean (SD)	70 (8.8)	68 (8.4)	69 (9.7)	71 (9.3)
Female	15,304 (54)	17,503 (59)	597 (59)	273 (59)
BMI mean (SD)	30.4 (5.2)	29.3 (4.8)	30.1 (5.7)	29.6 (5.9)
ASA				
1	2,281 (8.1)	3,938 (13)	61 (6.0)	10 (2.2)
2	21,086 (75)	19,435 (66)	738 (73)	313 (68)
3–4	4,863 (17)	6,273 (21)	213 (21)	134 (29)
OKS, mean (SD)				
preoperatively	19.4 (7.7)	23.7 (7.5)	22.8 (7.3)	19.7 (7.5)
valid score, n	27,990	29,648	1,015	457
6 months	36.9 (9.0)	38.0 (7.7)	35.5 (8.4)	–
valid score, n	27,835	25,349	405	
12 months	–	39.3 (7.8)	37.3 (8.7)	36.0 (8.9)
valid score, n		29,624	988	459

a English: United Kingdom National Joint Registry (NJR).

b Dutch: Dutch Arthroplasty Register (LROI).

c Danish: Copenhagen University Hospital Hvidovre’s local arthroplasty database.

d French (Swiss): Genova Arthroplasty Registry.

OHS: Oxford Hip Score, OKS, Oxford Knee Score, SD: standard deviation, BMI: body mass index, ASA: American Society of Anesthesiologists physical status classification system, TKA: total knee arthroplasty, UKA: unicompartmental knee arthroplasty.

### Outcomes

#### OHS

*Unidimensionality:* Separate language cohort evaluations of structural validity including CFA performed on OHS datasets showed higher than the suggested RMSEA threshold for good model fit for all language cohorts, but good SRMR, CFI, and TLI across all cohorts (Table 2).

**Table 2:** Confirmatory factor analysis of the OHS and OKS on preoperative data, performed on each cohort separately

CFA fit ^a^	English	Dutch	Danish	French (Swiss)
**OHS**, n	20,933	36,792	815	588
RMSEA	0.072	0.091	0.092	0.076
CI	0.070–0.073	0.090–0.092	0.084–0.100	0.066–0.086
SRMR	0.049	0.059	0.067	0.055
CFI	0.990	0.984	0.981	0.991
TLI	0.988	0.980	0.977	0.989
**OKS**, n	27,990	29,648	1,015	457
RMSEA	0.061	0.059	0.057	0.060
CI	0.060–0.062	0.057–0.060	0.050–0.065	0.048–0.072
SRMR	0.044	0.042	0.049	0.053
CFI	0.991	0.917	0.990	0.989
TLI	0.989	0.899	0.987	0.987

CI: 95% confidence interval, OHS: Oxford Hip Score, OKS, Oxford Knee Score, CFA: confirmatory factor analysis, RMSEA; Root Mean Square Error of Approximation, SRMR: Standardized Root Mean Square Residual, CFI: Comparative Fit Index, TLI: Tucker–Lewis Index.

a Adequate CFA model fit was defined as RMSEA < 0.06, SRMR < 0.08, CFI > 0.95, and TLI > 0.95.

*Monotonicity:* The items showed evidence of a monotonic relationship, with Loevinger’s Hi statistic >0.3 for all items (Supplementary Table S1).

*Item independence:* We detected indications of item dependence (Yen Q3 value > 0.2) between “washing” and “dressing” (Yen Q3: 0.271 for English, 0.358 for Dutch, 0.399 for Danish, and 0.265 for French cohorts) and between “sudden pain” and “night pain” (Yen Q3: 0.264 for English, 0.259 for Dutch, 0.304 for Danish, and 0.230 for French cohorts) across all language cohorts, and additionally between “pain” and “sudden pain” (Yen Q3: 0.206), “pain” and “night pain” (Yen Q3: 0.208), and “standing” and “night pain” (Yen Q3: 0.220) for the Danish cohort, and between “limping” and “sudden pain” (Yen Q3: 0.216) for the French cohort.

*Cross-cultural measurement invariance:* For the OHS, the multiple-group CFA performed on the full, matched (Supplementary Table S3) and random sample datasets, the overall fit of the models was not optimal (RMSEA > 0.10). However, the CFI changed 0.012, 0.011, and 0.012 between baseline (configural) models and threshold invariance models for all the full, matched, and random sample datasets, while RMSEA changed less than 0.015, or improved between gradually more constrained models, indicating measurement invariance between language cohorts (Table 3).

**Table 3:** Multi-group confirmatory factor analysis across English, Dutch, Danish, and French language cohorts for the Oxford Hip Score performed on full datasets, matched datasets, and random samples of 588 observations

Model constraint	Chi-square	df	P value	RMSEA ^a^	CFI	TLI ^a^
Full datasets						
Baseline						
model	40,679	216	< 0.001	0.113	0.946	0.934
Threshold invariance						
model	50,195	288	< 0.001	0.108	0.934	0.939
Threshold and loading invariance						
model	39,952	321	< 0.001	0.091	0.947	0.957
Matched datasets						
Baseline						
model	1,712	216	< 0.001	0.109	0.951	0.940
Threshold invariance						
model	2,098	285	< 0.001	0.104	0.940	0.945
Threshold and loading invariance						
model	1,818	318	< 0.001	0.090	0.950	0.959
Random sample datasets						
Baseline						
model	1,828	216	< 0.001	0.113	0.948	0.937
Threshold invariance						
model	2,264	288	< 0.001	0.108	0.937	0.942
Threshold and loading invariance						
model	1,951	321	< 0.001	0.093	0.948	0.957

df: degrees of freedom, RMSEA: Root Mean Square Error of Approximation, CFI: Comparative Fit Index, TLI: Tucker–Lewis Index.

a RMSEA changing ≤ 0.015, together with CFI changing < 0.01 between the 3 models indicated invariance across languages.

#### OKS

*Unidimensionality:* The RMSEA was only slightly higher than the suggested threshold for the English-language OKS cohort, while the CFI and TLI were lower than the thresholds for the Dutch cohort (see Table 2).

*Monotonicity:* Across all cohorts, all items showed evidence of monotonicity besides night pain for the Dutch cohort (Loevinger’s Hi statistic 0.294) and walking for the French cohort (Loevinger’s Hi statistic 0.277) (Supplementary Table S2).

*Item independence:* Item dependence was indicated between “washing” and “transport” for the English (Yen Q3: 0.245) and French (Yen Q3: 0.261) cohorts and between “pain” and “standing” for the French cohort (Yen Q3: 0.202).

*Cross-cultural measurement invariance:* For the OKS, the multiple-group CFA models across the full, matched (Supplementary Table S3) and random sample datasets likewise provided no strong evidence of non-invariance across language versions in the measurement models with change in CFI < 0.01 and change in RMSEA < 0.015 (Table 4).

**Table 4:** Multi-group confirmatory factor analysis across English, Dutch, Danish, and French language cohorts for the Oxford Knee Score performed on full datasets, matched datasets, and random samples of 457 observations

Model constraint	Chi-square	df	P value	RMSEA ^a^	CFI	TLI ^a^
Full datasets						
Baseline						
model	19,912	216	< 0.001	0.079	0.965	0.958
Threshold invariance						
model	24,887	288	< 0.001	0.076	0.957	0.960
Threshold and loading invariance						
model	20,827	321	< 0.001	0.066	0.964	0.970
Matched datasets						
Baseline						
model	908	216	< 0.001	0.084	0.960	0.951
Threshold invariance						
model	1,149	288	< 0.001	0.081	0.950	0.954
Threshold and loading invariance						
model	1,100	321	< 0.001	0.073	0.955	0.963
Random sample datasets						
Baseline						
model	918	216	< 0.001	0.084	0.960	0.951
Threshold invariance						
model	1,153	282	< 0.001	0.082	0.950	0.954
Threshold and loading invariance						
model	1,040	315	< 0.001	0.071	0.959	0.965

See footnote Table 3.

## Discussion

This is the first study to evaluate the cross-cultural validity of the English, Dutch, Danish, and French (Swiss) versions of the OHS and OKS. We used the recommended multiple-group CFA approach, in line with COSMIN guidelines, with a suitable number of observations [14]. We showed that, for both the OHS and OKS, structural validity was overall acceptable in each language version. When testing unidimensionality of the OHS, the CFA model fit was not optimal. Furthermore, we identified items that seemed to be dependent on other items. The multiple-group CFA for both OHS and OKS showed that the differences in factor structure and item thresholds (i.e., how respondents use the response categories) across the language versions were within predefined acceptable limits (i.e., based on changes in RMSEA and TLI). This suggests that the construct (hip or knee joint health) is measured invariantly across language versions and that group comparisons of latent means (and, with some caution, observed total scores) are meaningful.

The structural validity was not perfect, but acceptable. CFA results showed RMSEA model fit indices not meeting suggested model fit thresholds, which is in line with previous factor structure results of the English versions [21,22]. We did not identify studies using CFA to evaluate the structural validity in other language versions. The not optimal fit in OHS could suggest that hip pain and function cannot be considered a one-dimensional construct; however, more plausible explanations are that poorer fit may be driven by some response options that are less seldom used and overlap between items [18,23]. In support of this explanation, we found evidence of item dependence in 6 items in the OHS, with some variations across language versions. In all languages, there was dependence between washing and dressing, which is plausible, as both require similar functional hip movements and between sudden pain and night pain, which is also plausible because patients can experience sudden pain during the night. The not optimal model fit may translate into poorer discrimination, making it more difficult to separate patients with different levels of hip/knee health based on their scores [24]. However, considering their established use, it was not an aim to suggest other combinations of items or scoring of the OHS and OKS. Such modifications would come at the cost of reduced comparability with existing literature. The multiple-group CFA, our primary approach to evaluating the measurement invariance across language versions, showed baseline models with higher than suggested RMSEA cut-offs for good model fit, especially for the OHS (RMSEA > 0.1 for all multiple-group CFA models). However, other fit indices (CFI and TLI) were better. This model misfit can result from residual correlations (some items overlap), as were also found in the individual cohort CFAs. The results from testing the gradually more constrained models provided evidence of invariance between the 4 language versions. Considering the complex ordinal scale model and the large number of observations included [23], we view the absolute misfit depicted by the RMSEA levels as acceptable and interpret the results as supporting evidence of measurement invariance. In other words, the scales seem to measure the same construct in the same metric.

### Limitations

The results must be interpreted in relation to evidence of content validity, which is limited for non-English versions [14]. The English versions of the OHS and OKS were developed with input from patients to ensure the relevance, comprehensibility, and comprehensiveness of the selected questions [1,2]. Publications describing the subsequent translations, however, provide sparse information on whether patients were involved in evaluating content validity [6-10]. Within COSMIN standards, content validity is considered the most important measurement characteristic, as all other psychometric properties are reliant on it [25]. A recent Danish study found that the OHS questions covers aspects that are considered important and relevant to patients, but lack aspects such as certain physical activities, psychological health, and the hip problem’s impact on health-related quality of life [11]. While pointing to a need to evaluate other constructs, the results support the content validity of the 12-item OHS capturing hip health (pain and function combined). Furthermore, there were quite large differences in patient characteristics across cohorts, which could question whether the measurement properties reflect true language effects or rather differences in demographic characteristics. However, the multiple-group CFA fit indices performed on matched data were largely similar to those performed on unmatched data. Observed differences in descriptive scores are therefore more likely due to sample composition than to translation artifacts (i.e., patients from the UK and Switzerland had on average more severe symptoms than patients in the Netherlands and Denmark). However, residual non-invariance cannot be ruled out as there may have been other relevant matching factors. Differences in urbanicity may have introduced variability unrelated to language or cultural factors, given that the Swiss and Danish samples were not nationally representative. This may have reduced comparability between countries. Furthermore, and importantly, we included data from only 4 countries, all from Western Europe, and it is questionable how generalizable these results are to other language versions and countries. Lastly, we used a classical test theory approach to evaluate measurement invariance, at the level of the whole scale. Using item response theory approaches to detect differential item functioning can be used to detect variances on the item level. Such analyses are relevant to explore the found item dependence.

### Conclusion

The OHS and OKS demonstrated measurement invariance across the 4 language versions with multiple-group CFA model fit within predefined acceptable limits. In perspective, our findings support comparisons of the OHS and OKS across the included language versions in patients undergoing hip or knee arthroplasty.

### Supplementary data

Supplementary Figures 1–2 and Tables 1–3 are available as supplementary data on the article page, doi: 10.2340/17453674.2026.46545

## Supplementary Material



## Notes

LHI: conceptualization (lead); methodology (equal); writing—original draft (lead); formal analysis (lead). SAS: resources (equal); review and editing (equal), formal analysis (equal), methodology (equal). EB: conceptualization (supporting); review and editing (equal). KBC: methodology (equal); review and editing (equal). AJP, AL, ASS, AT, CB: resources (equal); review and editing (equal). JM: review and editing (equal). CH: methodology (equal); review and editing (equal). JMW: resources (equal); conceptualization (supporting); review and editing (equal).

The ISAR PROMs working group provided input to the study protocol. The authors would like to thank the participating registries for contributing data for this study.

Handling co-editor: Li Felländer-Tsai.

*Acta* thanks Anne Garland and Margareta Hedström for help with peer review of this manuscript.
