# Supplementary material for: The Oxford Hip and Knee Scores in patients undergoing hip and knee arthroplasty: cross-cultural validation study based on 110,000 patients
Source: Acta Orthop. 2026 Sep 3;97:602–8. doi: 10.2340/17453674.2026.46545 (PMC13543272; doi:10.2340/17453674.2026.46545)
Supplement: Supplementary file 1 [file ActaO-97-46545-s1.pdf]

Supplementary data

Cross-cultural validity of the Oxford Hip and Knee Scores in patients undergoing hip and knee arthroplasty

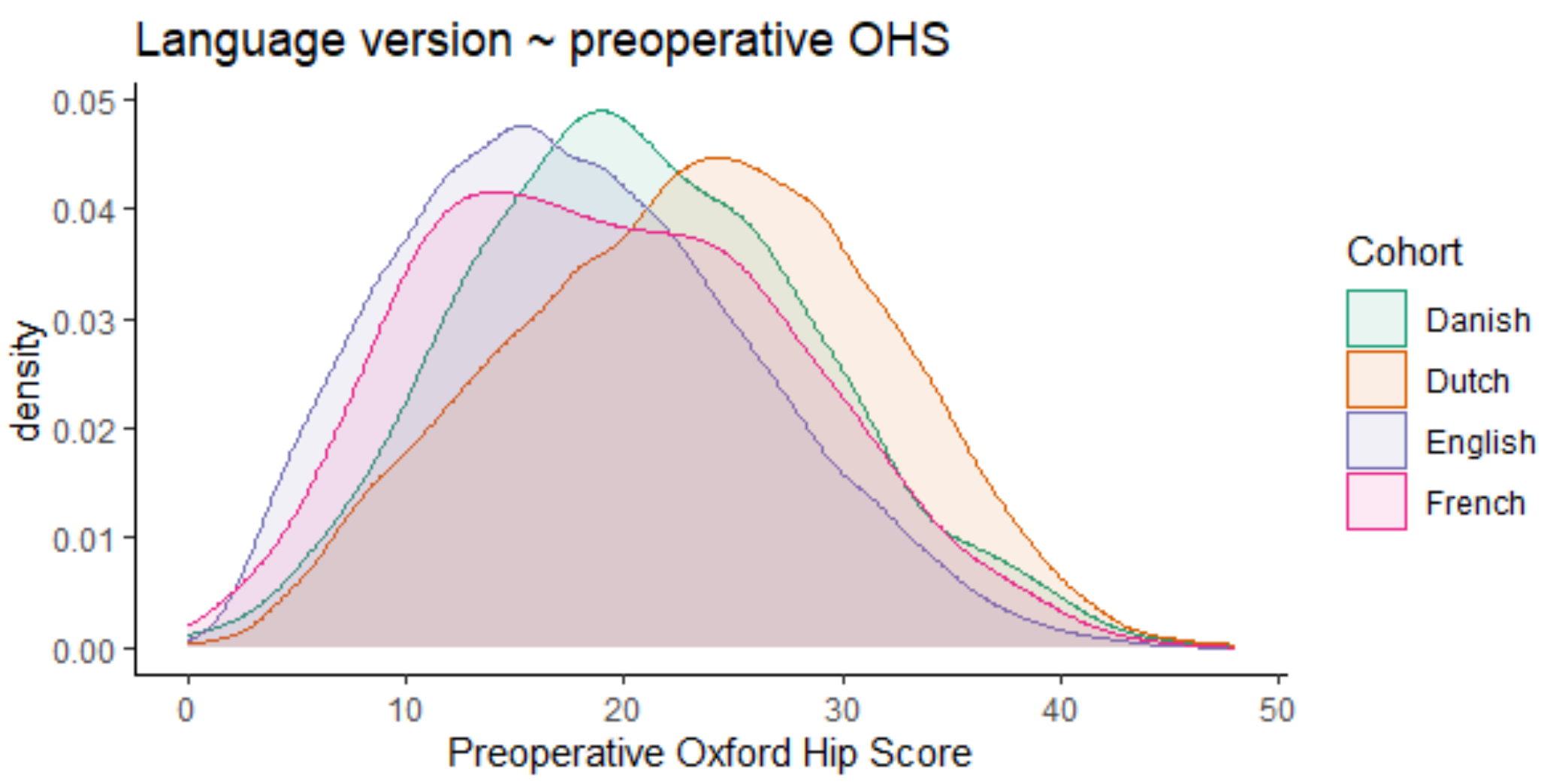

Figure S1. Density plot of preoperative Oxford Hip Scores across language cohorts. Curves represent kernel density estimates of the distributions; higher peaks indicate the most common values, and the degree of overlap reflects similarity between groups.

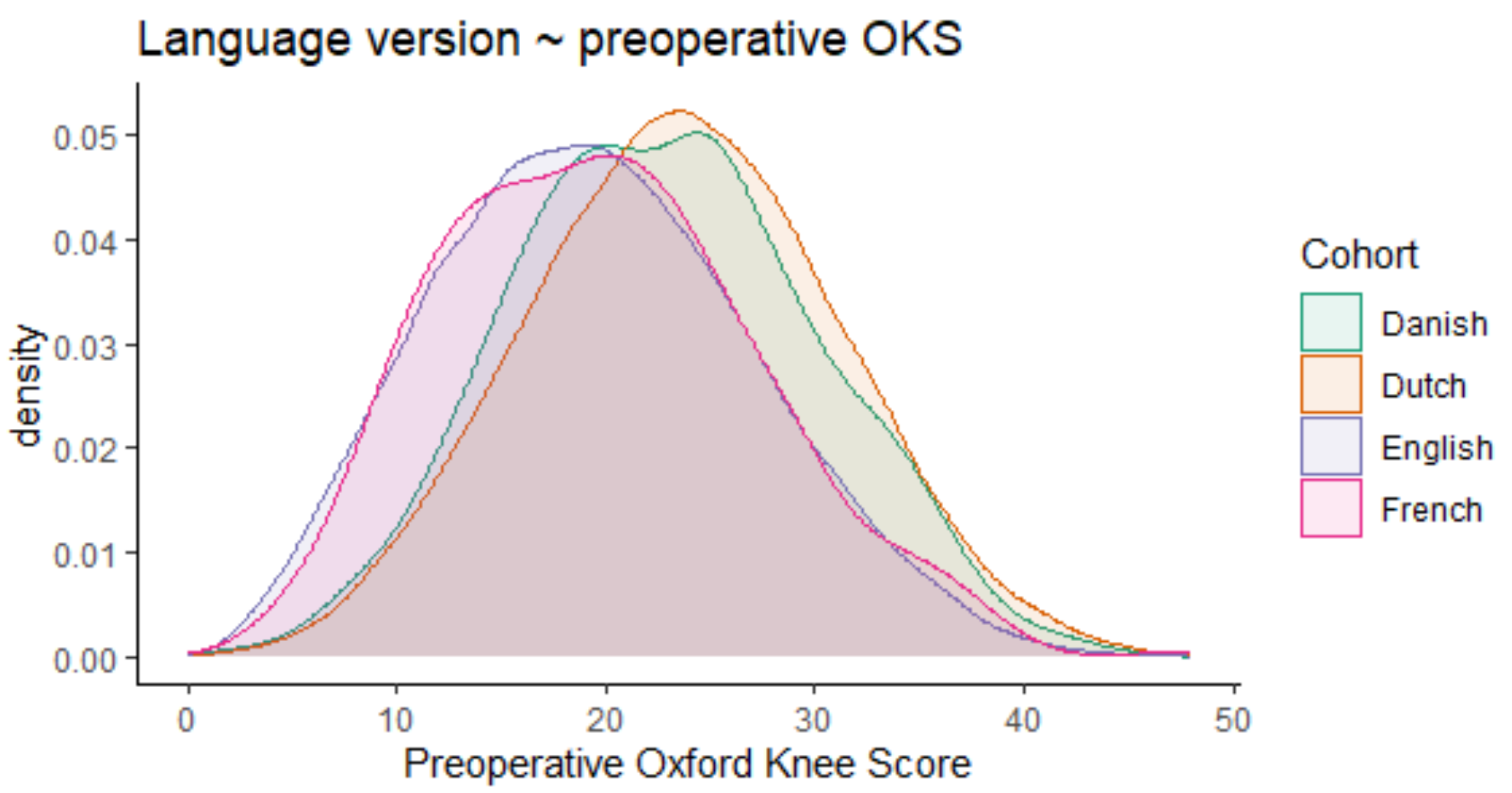

Figure S2. Density plot of preoperative Oxford Knee Scores across language cohorts. Curves represent kernel density estimates of the distributions; higher peaks indicate the most common values, and the degree of overlap reflects similarity between groups.

Table S1. Monotonicity: Loevinger's H statistics for each item in the Oxford Hip Score across language cohorts. Loevinger's H_i coefficient > 0.3 were considered to reflect monotonicity.

**OHS item**	**English**	**Dutch**	**Danish**	**French (Swiss)**
1. Pain	0.427	0.462	0.435	0.479
2. Washing	0.459	0.474	0.419	0.493
3. Transport	0.640	0.487	0.469	0.524
4. Dressing	0.570	0.428	0.400	0.462
5. Shopping	0.603	0.504	0.471	0.549
6. Walking	0.566	0.414	0.386	0.417
7. Stairs	0.646	0.493	0.462	0.551
8. Standing up	0.647	0.495	0.449	0.518
9. Limping	0.576	0.331	0.358	0.442
10. Sudden pain	0.520	0.372	0.326	0.482
11. Work	0.669	0.533	0.512	0.579
12. Night pain	0.540	0.337	0.330	0.384

Table S2. Monotonicity: Loevinger's H statistics for each item in the Oxford Knee Score across language cohorts. Loevinger's H_i coefficient > 0.3 were considered to reflect monotonicity.

**OKS item**	**English**	**Dutch**	**Danish**	**French (Swiss)**
1. Pain	0.459	0.434	0.409	0.442
2. Washing	0.431	0.395	0.385	0.358
3. Transport	0.481	0.441	0.428	0.448
4. Walking	0.405	0.340	0.344	0.277
5. Standing up	0.491	0.459	0.414	0.417
6. Limping	0.387	0.347	0.318	0.369
7. Kneeling	0.426	0.355	0.360	0.402
8. Night pain	0.375	0.294	0.308	0.348
9. Work	0.540	0.467	0.464	0.483
10. Give way	0.427	0.327	0.326	0.322
11. Shopping	0.508	0.470	0.462	0.483
12. Stairs	0.503	0.451	0.420	0.442

Table S3. Standardized mean differences before and after matching English, Dutch and Danish data to the French (Swiss) datasets for OHS (n = 588) and OKS (n = 457) analyses, separately.

Covariate	English before	English after	Dutch before	Dutch after	Danish before	Danish after
**OHS**						
Age	0.067	0.000	0.078	0.000	-0.086	0.026
BMI	0.174	0.000	-0.052	0.000	0.093	0.032
ASA	-0.255	0.000	-0.196	0.000	-0.144	-0.019
Sex	-0.084	0.000	-0.174	0.000	-0.046	0.010
**OKS**						
Age	-0.051	0.000	-0.132	0.000	-0.218	0.000
BMI	0.089	0.000	-0.049	0.000	0.074	0.000
ASA	-0.273	0.000	-0.185	0.000	-0.182	0.000
Sex	0.107	0.000	0.010	0.000	0.016	0.000

OHS: Oxford Hip Score, OKS, Oxford Knee Score, BMI: body mass index, ASA: American Society of Anesthesiologists physical status classification system
